# Hepatic immunophenotyping for streptozotocin-induced hyperglycemia in mice

**DOI:** 10.1038/srep30656

**Published:** 2016-07-28

**Authors:** Young-Sun Lee, Hyuk Soo Eun, So Yeon Kim, Jong-Min Jeong, Wonhyo Seo, Jin-Seok Byun, Won-Il Jeong, Hyon-Seung Yi

**Affiliations:** 1 Department of Internal Medicine, Korea University College of Medicine, Seoul 136-705, Republic of Korea; 2 Department of Internal Medicine, Chungnam National University School of Medicine, Daejeon 305-764, Republic of Korea; 3 Laboratory of Liver Research, Biomedical Science and Engineering Interdisciplinary program, Korea Advanced Institute of Science and Technology, Daejeon 34141, Republic of Korea; 4 Department of Oral Medicine, School of Dentistry, Kyungpook National University, Daegu 41566, Republic of Korea; 5 Laboratory of Liver Research, Graduate School of Medical Science and Engineering, Korea Advanced Institute of Science and Technology, Daejeon 34141, Republic of Korea

## Abstract

Emerging evidence revealed that diabetes induces abnormal immune responses that result in serious complications in organs. However, the effect of hyperglycemia on hepatic immunity remains obscure. We evaluated the population and function of hepatic immune cells in streptozotocin (STZ)-induced hyperglycemic mice. CC chemokine receptor 2 (CCR2)-knockout mice and mice with a depletion of regulatory T cells (DEREG) were used to investigate the migration and role of regulatory T cells (Tregs) in hyperglycemic mice. The inflammatory cytokines and hepatic transaminase levels were significantly increased in the hyperglycemic mice. The population and number of infiltrating monocytes, granulocytes, and Tregs were enhanced in the livers of the hyperglycemic mice. Hepatic monocytes other than macrophages showed the increased expression of inflammatory cytokines and chemokines in the hyperglycemic mice. The CCR2 knockout and DEREG chimeric mice exhibited increased populations of activated T cells and neutrophils compared to the WT chimeric mice, which promoted hepatic inflammation in the hyperglycemic mice. The migration of CCR2 knockout Tregs into the liver was significantly reduced compared to the WT Tregs. We demonstrated that hyperglycemia contributes to increase in infiltrating monocytes and Tregs, which are associated with hepatic immune dysfunction in mice. CCR2-mediated migration of Tregs regulates hyperglycemia-induced hepatic inflammation.

Diabetes mellitus has been predicted to produce inflammatory cytokines and induce the activation of innate and adaptive immune cells, which are associated with diabetic complications^1^,^2^,^3^. The liver is an essential part of the innate immune response and is composed of diverse types of immune cells, including monocytes, macrophages, lymphocytes, natural killer cells, and hepatic stellate cells (HSCs)^4^,^5^. Nonalcoholic fatty liver disease including simple steatosis and nonalcoholic steatohepatitis is the predominant hepatic manifestations of diabetes and are considered the most important causes of liver injury^6^,^7^. However, the mechanisms that contribute to liver damage in patients with diabetes and hyperglycemia remain obscure.

Immune cells participate in the development of systemic inflammation in hyperglycemic patients. Monocytes from patients with diabetes have shown higher expression of pro-inflammatory cytokines, including interleukin (IL)-1 beta, IL-6, and tumor necrosis factor alpha (TNF-α)^8^,^9^. Moreover, B cells activate T-cell-mediated systemic inflammation and decrease anti-inflammatory cytokine IL-10 expression in obese mice and patients with type 2 diabetes, respectively^10^,^11^, and T cells promote chronic inflammation in diabetes patients through interferon gamma (IFN-γ) and IL-17 production^12^. Conversely, CD4^+^ CD25^+^ Foxp3^+^ regulatory T cells (Tregs) inhibit T cell-mediated pathology by producing IL-10 in the adipose tissue of obese mice^13^,^14^. The loss of hepatic Tregs has been implicated as an important marker for the aggravation of liver inflammation^15^,^16^. However, the effects of hyperglycemia on Tregs in the liver are unknown.

Non-obese diabetic (NOD) mice are commonly used as an animal model for type 1 diabetes. In this study, hyperglycemia was induced in mice with multiple low doses (50 mg/kg) of streptozotocin (STZ) for five consecutive days. Because NOD mice have immunological alterations due to genetic defects and are susceptible to multi-organ autoimmune diseases other than autoimmune diabetes via the production of autoreactive T cells^17^,^18^, we used STZ-induced hyperglycemic mice to investigate whether hyperglycemia had an effect on the population and activation of hepatic immune cells. STZ is the most widely used chemical for the generation of type 1 diabetes phenotypes through the destruction of insulin-producing cells in mice. STZ is a toxic nitrosourea analogue that targets pancreatic beta cells via glucose transporter 2 in mice and rats^19^,^20^. In the present study, we investigated whether STZ-induced hyperglycemia led to hepatic inflammation and changes in the population size and function of the immune cells in the liver. Additionally, we investigated the importance of Tregs in hepatic immune homeostasis through the regulation of Treg depletion or migration to the liver.

## Materials and Methods

### Animals

Male C57BL/6 wild-type (WT), green fluorescence protein (GFP)-transgenic, CC chemokine receptor 2 (CCR2) knockout, and depletion of regulatory T cell (DEREG) mice were purchased from the Jackson Laboratory (Bar Harbor, ME, USA). All animals were maintained under a controlled environment (12 h light/12 h dark cycle; humidity 50~60%; ambient temperature 22 ± 2 °C) in a specific pathogen-free animal facility at the Korea Advanced Institute of Science and Technology. Chimeric mice were prepared by bone marrow transplantation as previously reported^21^. All animals received humane care according to the criteria outlined in the Guide for the Care and Use of Laboratory Animals published by the National Institutes of Health, and all experimental procedures were approved by the Institutional Animal Care and Use Committee of the Korea Advanced Institute of Science and Technology.

### Materials

STZ was purchased from Sigma (St. Louis, MO, USA). The total RNA isolation kits and reagents for cDNA synthesis were obtained from Thermo Scientific (Invitrogen, Eugene, OR, USA). The Percoll gradient and DNase I were obtained from GE Healthcare (Buckinghamshire, UK) and Roche (Indianapolis, IN, USA), respectively. The SYBR Green Real-Time PCR Master Mix was purchased from Toyobo (Osaka, Japan). Antibodies were obtained from Abcam (Cambridge, MA, USA), BD Pharmingen (CA, USA) and eBioscience (CA, USA). The FlowJo software was purchased from Tree Star (Ashland, OR, USA).

### STZ-induced hyperglycemia

Hyperglycemia was induced in mice by intraperitoneal injection of multiple low doses (50 mg/kg) of STZ solubilized in 0.1 M citrate buffer (pH 4.5) for five consecutive days. Control mice were injected with the same volume of citrate buffer alone. The mice were monitored by measurements of body weight, food and water intake, blood glucose, and serum insulin 4 days after the final STZ injection by obtaining blood from the tail vein. To exclude the effect of STZ toxicity on the immune cell population in the liver, STZ mice were injected with subcutaneous Humulin N (Ely Lilly, USA) at a dose of 50 IU/kg bodyweight. Moreover, the mice with STZ-induced hyperglycemia received an intraperitoneal injection of anti-mouse CCR2 antibodies (clone MC-21, 50 ug/day) every other day until sacrifice.

### Serum biochemical measurements

Blood was collected and allowed to clot at room temperature for 1 hour and then centrifuged at 8,000 rpm for 10 minutes. Serum was removed and stored at −20 °C. The serum alanine aminotransferase (ALT), aspartate aminotransferase (AST), total cholesterol (TC), triglyceride (TG), and glucose levels were measured using kits purchased from IDEXX Laboratories (Westbrook, ME, USA). Serum insulin was measured using the Ultrasensitive Mouse Insulin ELISA kit (Alpco, Salem, NH, USA).

### Enzyme-linked immunosorbent assay (ELISA)

Blood serum was added to a plate pre-coated with monoclonal antibodies against mouse IFN-γ (eBioscience, San Diego, CA, USA) or TNF-α (eBioscience, San Diego, CA, USA). After overnight incubation at 4 °C, an anti-mouse IFN-γ or TNF-α antibody conjugated to horseradish peroxidase was added and the plate was incubated for additional 1 additional hour at room temperature on the shaker. The color was developed with 50 μl of 3,3′5,5′-Tetramethylbenzidine substrate (eBioscience, San Diego, CA, USA). The reaction was stopped with the addition of 50 μl of 2N H_2_SO_4,_ and the absorbance of the reaction as measured at 450 nm using a microplate reader (Bio-Rad, Hercules, CA, USA).

### Isolation of mononuclear cells (MNCs) in the liver

Liver MNCs were isolated as described previously^21^. Briefly, liver tissues were smashed and filtered through a cell strainer with 70 μm nylon mesh (BD falcon, NJ, USA). The liver cell suspension was suspended in cold phosphate buffered saline (PBS), and hepatocytes were removed by centrifugation at 400 rpm for 5 minutes. The supernatant was collected, washed in PBS at least 2 times, and resuspended in a 40% Percoll gradient (GE healthcare, UK) in PBS. The cell suspension was centrifuged at 2,400 rpm at 4 °C for 30 minutes. The supernatant was removed with mechanical suction and then red blood cell lysis was performed. The MNCs were collected, washed in PBS, and resuspended in RPMI-1640 medium. Finally, the MNCs were counted and subjected to flow cytometry and real-time PCR.

### Fluorescence-activated cell sorting (FACS) analysis

Isolated liver MNCs were resuspended in DPBS containing 0.5% BSA and 0.05% sodium azide. After washing, the cells were pre-incubated with anti-mouse CD16/32 Fc blocker (BD Pharmingen, USA) prior to treatment with antibodies to block non-specific reactions. The cells were stained with fluorescence-conjugated anti-CD45, anti-CD3e, anti-CD4, anti-CD8, anti-CD11b, anti-CD25, anti-CD44, anti-CD69, anti-Ly6C, anti-Ly6G, and anti-F4/80 antibodies (BD Pharmingen, USA). The stained cells were analyzed using a BD™ LSR II Flow Cytometer (BD Pharmingen, USA) and the FlowJo software (Tree Star, Ashland, OR, USA). PE-conjugated anti-IFN-γ and TNF-α antibodies (BD Bioscience, USA) were used for intracellular staining. For intracellular cytokine staining, the cells were re-stimulated with phorbol-myristate acetate/ionomycin for 1 hour in addition to brefeldin A for 5 hours and then the cells were fixed and permeabilized using the BD Cytofix/Cytoperm kit (BD Pharmingen, USA).

### Sorting and staining of liver immune cells

Infiltrating monocytes and macrophages in the liver were sorted using the FACSAria™ II Cell Sorter (BD Bioscience, USA) based on marker expression. Infiltrating monocytes and macrophages sorted from the liver were attached to slides via a cytospin at 500 rpm for 5 minutes, fixed with 4% paraformaldehyde, permeabilized with 0.2% Triton-X (Sigma-Aldrich, St. Louis, MO, USA), and then stained with hematoxylin and eosin as previously described^22^.

### HSC and liver sinusoidal endothelial cells (LSEC) preparation

As described previously^21^,^23^, HSCs were isolated by *in situ* collagenase perfusion. After anesthesia using intraperitoneal injection of pentobarbital (30 mg/kg), the abdominal cavity was opened, and the liver was exposed for perfusion with collagenase type I through the portal vein. After filtering the cell suspension using a 70 μm nylon mesh (BD Falcon, NJ, USA), hepatocytes and non-parenchymal cells were separated by centrifugation at 500 rpm and 4 °C for 5 minutes. Then, the HSCs and LSEC were isolated by differential centrifugation on the OptiPrep (Sigma, St. Louis, MO, USA) density gradient at 3,000 rpm and 4 °C for 17 minutes. The HSC purity was assessed by flow cytometric analysis based on the vitamin A autofluorescence of the HSCs. LSEC was positively purified by labelling with anti-mouse CD 146 microbeads (Miltenyi Biotec, CA, USA) and was subjected to Real-time PCR analysis.

### Real-time PCR analysis

The real-time PCR analysis was performed and all primers used in this study are listed in Supplementary Table 1. Total RNA was extracted from liver cells or tissues using the TRIzol Reagent (Invitrogen, Eugene, OR, USA) in accordance with the manufacturer’s instructions. cDNA was synthesized from the same quantity of RNA with the amfiRivert cDNA Synthesis Master Mix (GenDEPOT, USA) following the manufacturer’s protocol. Real-time PCR was performed using the SYBR Green Real-Time PCR Master Mix (Toyobo, Japan) and analyzed on a CFX96 system (Bio-Rad, Hercules, CA, USA). The comparative Ct method was used to quantify transcripts normalized to β-actin expression. The results were analyzed with the ΔΔCt method. The values were expressed as the fold change compared with the control.

### Chimeric mice generation

To investigate the effects of CCR2 and Tregs in the hyperglycemia-induced hepatic inflammation, we produced chimeric mice by bone marrow transplantation using WT, CCR2 knockout, and DEREG mice. For development of chimerism, recipient WT mice were fed with the water supplemented with 1% penicillin/streptomycin for 7 days and then irradiated with 950 rads. After 6 hours, about 2 × 10^6^ bone marrow cells from donor mice were injected via tail vein sterilely. To confirm formation of chimerism, recepient mice were sacrificed at 8 weeks and subjected to FACS analysis. At 8 weeks after the transplantation of WT, CCR2 knockout, and DEREG bone marrow, the recipient mice were injected with 1 g of diphtheria toxin intraperitoneally for 5 days and then treated with multiple low doses (60 mg/kg) of intravenous STZ injection for three consecutive days. The mice were sacrificed for the immunophenotyping study of the liver on Day 20 of STZ injection.

### *Ex vivo* migration assay of Tregs using closed circulation

Age-matched (8–12 weeks old) male CD45.1^+^ mice with STZ-induced hyperglycemia were anesthetized by intraperitoneal pentobarbital injection, and the inferior vena cava (IVC) and portal vein were exposed by midline laparotomy. The portal vein and superior part of the IVC above the liver were catheterized. Each catheter line was connected with an infusion pump, and the opposite end of the line was placed in a tube filled with 2 × 10^6^ Tregs from WT or CCR2 KO mice suspended in RPMI medium supplemented with 10% serum and 1% penicillin/streptomycin. To maintain the same sinusoidal pressure in the liver, the flow rate was fixed at 2 ml/min. The mice and all equipments were maintained in a 37 °C incubator during the closed circulation process. After 2 hours of closed circulation, the liver was extracted, and the resident hepatic MNCs were isolated for FACS analyses.

### Statistical analysis

Data are presented as the means ± the standard deviation (SD). To compare values obtained from two or more groups, Student’s t test or one-way analysis of variance was performed. A value of P < 0.01 or 0.05 was considered statistically significant.

## Results

### STZ-induced hyperglycemia increases hepatic monocytes and neutrophils in mice

**Figure 1:**
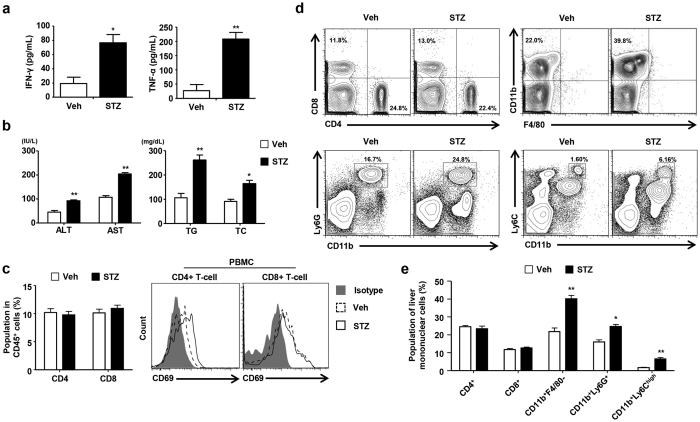
STZ-induced hyperglycemia increases monocytes and neutrophils in the mouse liver. Hyperglycemia was induced in C57BL/6 mice with low dose STZ (50 mg/kg) administration for five consecutive days. (**a**) IFN-γ, and TNF-α levels were measured in sera collected from mice treated with the vehicle or STZ. (**b**) AST, ALT, triglyceride, and TC were measured in sera collected from the mice. (**c**) T cell population and activation in mouse peripheral blood mononuclear cells. (**d**) Liver mononuclear cells were subjected to flow cytometry to analyze lymphocytes, monocytes, and neutrophils. (**e**) Percentages of hepatic immune cells in mice treated with the vehicle or STZ. All data are representative of 3 independent experiments (n = 6 per group). Data are expressed as the mean ± SD. *P < 0.05, **P < 0.01 compared with the corresponding controls.

We generated a mouse STZ-induced diabetes model to reveal the effects of hyperglycemia on the population characteristics and function of hepatic immune cells. Significant hyperglycemia (over 250 mg/dL) developed three or four days after the injection of multiple low-doses of STZ (Supplementary Fig. 1a–d). Mice were sacrificed 3 weeks after the induction of diabetes with STZ. The glucose, insulin, IFN-γ, and TNF-α levels were measured in sera collected from the control and diabetic mice. Serum IFN-γ and TNF-α levels were significantly increased in mice with STZ-induced hyperglycemia (Fig. 1a). Moreover, the blood chemistry analyses revealed that ALT, AST, TG, and TC levels were increased in mice with STZ-induced hyperglycemia (Fig. 1b). Hepatic TG was also enhanced in the livers from mice with STZ-induced hyperglycemia (Supplementary Fig. 1e). In the FACS analyses, we found that CD4^+^ T cells in the peripheral blood and mesenteric lymph nodes were significantly activated in the mice with STZ-induced hyperglycemia compared to the control mice, although there was no difference in the overall size of population and the absolute number of immune cells between the groups (Fig. 1c,d and Supplementary Fig. 2a–d). In contrast, the size of the monocytes (CD11b^+^ Ly6C^high^) and neutrophils (CD11b^+^ Ly6G^+^) populations in the livers from diabetic mice were higher than those from the control mice (Fig. 1d,e). A larger population of F4/80+ hepatic macrophages was observed in the sinusoidal compartment in mice with STZ-induced hyperglycemia (Supplementary Fig. 2e). Taken together, our results demonstrate that STZ-induced hyperglycemia enhances hepatic inflammatory response in the mice through an increase in monocytes and neutrophils.

### Migration of Tregs is enhanced in the livers of mice with STZ-induced hyperglycemia

**Figure 2:**
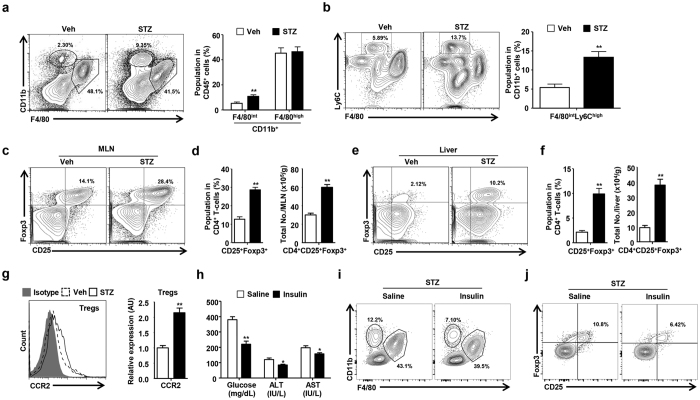
STZ-induced hyperglycemia promotes CCR2-mediated migration of Tregs in the liver. (**a**) The population of hepatic monocytes and macrophages in mice treated with the vehicle or STZ. (**b**) The population of hepatic monocytes in mice treated with the vehicle or STZ. (**c,d**) The population and absolute number of Tregs in the mesenteric lymph nodes of mice treated with the vehicle or STZ. (**e,f**) The population and absolute number of Tregs in the livers of mice treated with the vehicle or STZ. (**g**) CCR2 expression in hepatic Tregs from mice treated with the vehicle or STZ. (**h**) Glucose, AST, and ALT were measured in sera collected from the mice treated with saline or insulin. (**i,j**) The population of hepatic monocytes, macrophages, and Tregs in the STZ-induced hyperglycemic mice treated with the saline or insulin. All data are representative of 3 independent experiments (n = 6 per group). Data are expressed as the mean ± SD. *P < 0.05, **P < 0.01 compared with the corresponding controls.

Based on the above data, STZ-induced hyperglycemia changed the population of hepatic innate immune cells. We also isolated liver mononuclear cells using the perfusion technique to analyze hepatic infiltrating monocytes and liver resident macrophages for comparison with circulating monocytes. Infiltrating monocytes (CD11b^+^ F4/80^int^) of the liver were increased in the population and absolute number in mice with STZ-induced hyperglycemia, although the population of liver resident macrophages (CD11b^+^ F4/80^high^) did not change between the groups (Fig. 2a,b and Supplementary Fig. 2f). Tregs are essential for the modulation of tissue inflammation through the secretion of anti-inflammatory cytokines. As expected, the population and absolute number of the Treg were significantly increased in the mesenteric lymph nodes (Fig. 2c,d) and livers of the mice with STZ-induced hyperglycemia compared to the control mice (Fig. 2e,f). Interestingly, the expression of CCR2 on Tregs was significantly increased in mice with STZ-induced hyperglycemia compared to the control mice (Fig. 2g). To investigate whether these modulations of hepatic immune cells were caused by hyperglycemia or effect of STZ toxicity, the STZ treated mice were administered saline or insulin (Humulin N) at a dose of 50 IU/kg. We examined the effect of insulin-mediated reduction of blood glucose on the hepatic immune cell population in STZ-induced hyperglycemic mice. Insulin therapy reduced the levels of glucose, ALT, and AST in the mice with STZ-induced hyperglycemia (Fig. 2h). Infiltrating monocytes of the liver were also decreased in the population in the hyperglycemic mice with the insulin treatment (Fig. 2i). The population of the Treg were significantly decreased in the liver from the hyperglycemic mice treated with insulin (Fig. 2j). Furthermore, the treatment with CCR2 antibodies increased the level of hepatic transaminases and reduced the population of Treg in the liver from STZ-induced hyperglycemic mice (Supplementary Fig. 2g,h). Collectively, STZ-induced hyperglycemia induced liver inflammation through the activation of hepatic infiltrating monocytes, thereby promoting the recruitment of the Tregs into the liver.

### Gene expression of hepatic immune cells in mice with STZ-induced hyperglycemia

**Figure 3:**
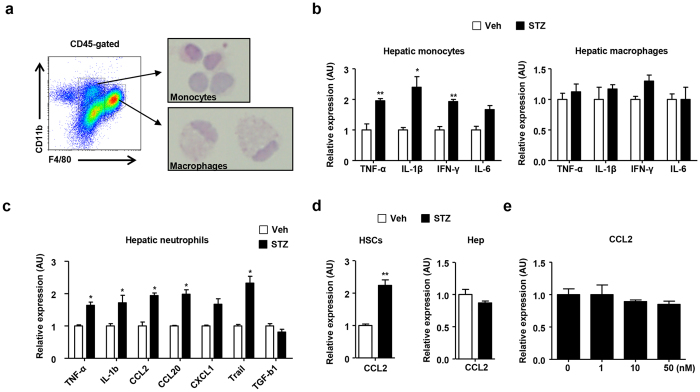
STZ-induced hyperglycemia enhances the expression of proinflammatory cytokines and chemokines in monocytes but not liver resident macrophages. (**a**) Morphological analysis of hepatic monocytes and macrophages using FACS. (**b**) Sorted hepatic monocytes and macrophages from mice treated with vehicle or STZ were subjected to real-time PCR analysis. (**c**) Sorted hepatic neutrophils from mice treated with vehicle or STZ were subjected to real-time PCR analysis. (**d**) HSCs and hepatocytes isolated from mice treated with vehicle or STZ were subjected to real-time PCR. (**e**) HSCs were treated with STZ *in vitro*. All data are representative of 3 independent experiments (n = 6 per group). Data are expressed as the mean ± SD. *P < 0.05, **P < 0.01 compared with the corresponding controls.

To assess the effect of hyperglycemia on hepatic infiltrating monocytes and macrophages, the cells were isolated using the fluorescence activated cell sorting cytometry. (Fig. 3a). The morphological features of the sorted monocytes and macrophages were observed after H&E staining. There was no significant difference in the morphologies of the liver monocytes and macrophages between the groups (Fig. 3a and Supplementary Fig. 3a). In the real-time PCR analysis, the infiltrating hepatic monocytes from diabetic mice showed remarkably increased expression of TNF-α, IL-1β, IFN-γ, and IL-6 compared with the control mice (Fig. 3b). However, there was no significant difference in the expression of these inflammatory cytokines between the groups of liver resident macrophages (Fig. 3b). In contrast, neutrophils from the diabetic mice showed enhanced expression of IL-1β and TNF-α compared with the control mice (Fig. 3c). We also isolated hepatocytes and HSCs from the diabetic and control mice to observe the expression of CC chemokine ligand 2 (CCL2), which is a major ligand of CCR2. Although there was no significant difference in CCL2 expression on the hepatocytes and LSEC between groups, HSCs from diabetic mice showed increased expression of CCL2 compared with the control mice (Fig. 3d and Supplementary Fig. 3b). To assess the effect of STZ *in vitro*, we directly treated the HSCs with STZ. However, treatment with STZ did not have an effect on CCL2 expression in the HSCs (Fig. 3e). Moreover, there was no significant difference in the expression of IL-6, IFN-γ, and IL-10 expression in the HSCs after STZ treatment (Supplementary Fig. 3c).

### CCR2 and Tregs are important for the regulation of hyperglycemia-induced hepatic inflammation

**Figure 4:**
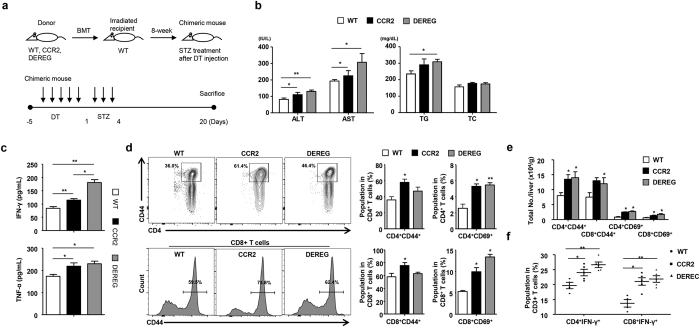
CCR2-mediated migration of Tregs regulates hyperglycemia-induced hepatic inflammation. (**a**) At week 8 after the transplantation of WT, CCR2 KO, and DEREG bone marrow into WT mice, the chimeric mice were injected with diphtheria toxin and STZ. (**b**) AST, ALT, triglyceride, and TC levels were measured in sera collected from the mice. (**c**) IFN-γ and TNF-α were measured in sera collected from the three groups of mice. (**d,e**) Hepatic T cell population and absolute number in the three groups of mice. (**f**) IFN-γ^+^ T cell population in the livers of in WT, CCR2 knockout, and DEREG mice. All data are representative of 3 independent experiments (n = 6–8 per group). Data are expressed as the mean ± SD. *P < 0.05, **P < 0.01 compared with the corresponding controls.

To investigate the role of Tregs in hyperglycemia-induced hepatic inflammation, we generated chimeric mice by reciprocal bone marrow transplantation using WT, CCR2 KO, and DEREG mice. Approximately 90% of the liver MNCs were replaced with donor bone marrow-derived cells 8 weeks after the transplantation of WT GFP-expressing bone marrow (Supplementary Fig. 4a). Chimeric mice prepared by reciprocal transplantation of WT, CCR2 KO, or DEREG bone marrow were injected with 1 μg of DT diluted in endotoxin-free PBS for five consecutive days. On day 6, the three groups of chimeric mice treated with DT were subjected to STZ treatment. All of the chimeric mice were sacrificed for the analysis of serum chemistry and hepatic immune cells on day 20 (Fig. 4a). No significant difference was detected in blood glucose levels and food and water intake among the three groups (Supplementary Fig. 4b–d). ALT, AST, and TG levels were significantly increased in the mice injected with the CCR2 KO or DEREG bone marrow compared to the mice injected with the WT bone marrow (Fig. 4b). Moreover, the IFN-γ and TNF-α levels were increased in the mice injected with the CCR2 KO or DEREG bone marrow (Fig. 4c). The hepatic TG content was enhanced in the mice injected with the DEREG bone marrow compared to the mice injected with the WT bone marrow (Supplementary Fig. 4e). In the FACS analysis of the liver MNCs, the populations of activated CD4^+^ and CD8^+^ T cells were increased in the population and absolute number of the hepatic immune cells in the mice injected with the CCR2 KO or DEREG bone marrow (Fig. 4d,e and Supplementary Fig. 5). The hepatic CD4^+^ IFN-γ^+^ and CD8^+^ IFN-γ^+^ T cell populations were also significantly increased in the CCR2 KO and DEREG chimeric mice (Fig. 4f). Taken together, our results suggested that the depletion of Tregs or CCR2 deficiency induced hepatic inflammation in STZ-induced hyperglycemic mice.

### CCR2 is critical for the migration of Tregs into the livers of hyperglycemic mice

**Figure 5:**
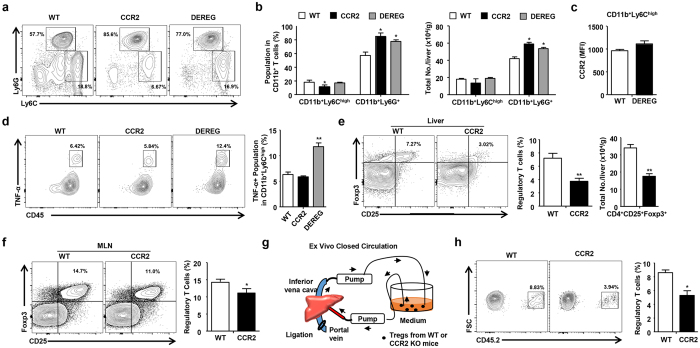
CCR2 is critical for the migration of Tregs into the livers of hyperglycemic mice. (**a,b**) The population and absolute number of hepatic monocytes and neutrophils. (**c**) CCR2 expression in hepatic monocytes from WT and DEREG mice. (**d**) FACS analysis of infiltrating hepatic monocytes in WT, CCR2 knockout, and DEREG mice. (**e**) Tregs in the livers of WT and CCR2 chimeric mice. (**f**) Tregs in the mesenteric lymph nodes of WT and CCR2 chimeric mice. (**g**) Schematic model for the *ex vivo* closed circulation method. (**h**) Migration assay of WT or CCR2 KO Tregs using the *ex vivo* closed circulation methods with 4 independent experiments. All data excluding closed circulation experiements are representative of 3 independent experiments (n = 6–8 per group). Data are expressed as the mean ± SD. *P < 0.05, **P < 0.01 compared with the corresponding controls.

Next, we investigated whether STZ-induced hyperglycemia was responsible for the population of inflammatory monocytes and neutrophils in the three groups of chimeric mice. The neutrophil population was significantly increased in mice injected with CCR2 KO or DEREG bone marrow although there was no increase in the monocyte population in mice transplanted with CCR2 KO or DEREG bone marrow (Fig. 5a,b). CCR2 expression in hepatic monocytes was not changed between WT and DEREG chimeric mice (Fig. 5c). The infiltrating monocytes and neutrophils in the liver were increased in the mice transplanted with the CCR2 KO or DEREG bone marrow, although the population of liver resident macrophages (CD11b^+^ F4/80^high^) did not change between the groups (Supplementary Fig. 6a,b). The neutrophils from the CCR2 KO or DEREG chimeric mice with hyperglycemia showed enhanced expression of IL-1β and TNF-α compared with those from the control mice (Supplementary Fig. 6c). Moreover, TNF-α expression in the infiltrating hepatic monocytes was enhanced in the DEREG chimeric mice with hyperglycemia compared to the WT or CCR2 KO chimeric mice with hyperglycemia (Fig. 5d). IL-10, Foxp3, and transforming growth factor beta 1 expression was significantly down-regulated in the Tregs isolated from the CCR2 KO chimeric mice (Supplementary Fig. 6d). Moreover, the population and absolute number of Tregs were significantly reduced in the liver and mesenteric lymph nodes from the CCR2 chimeric mice compared to the WT chimeric mice (Fig. 5e,f and Supplementary Fig. 6e). To investigate the Treg migration, the Tregs isolated from the WT or CCR2 KO mice were circulated in RPMI medium supplemented with 10% serum for 2 hours through a closed circulation system from the portal vein to the inferior vena cava in CD45.1^+^ mice with STZ-induced hyperglycemia (Fig. 5g). The migration of Tregs was significantly reduced in the circulation with Tregs from the CCR2 KO mice compared to those from the WT mice (Fig. 5h). These data demonstrate that CCR2-mediated migration of Tregs is important for the regulation of hepatic inflammation in mice with STZ-induced hyperglycemia.

## Discussion

In the present study, we demonstrated that CD11b^+^ Ly6C^high^ inflammatory monocytes and CD11b^+^ Ly6G^+^ neutrophils were significantly elevated in the livers of mice with STZ-induced hyperglycemia, although there was no difference in the population size and function of liver resident macrophages between groups. Recently, hyperglycemia was shown to lead to the activation of human monocytes via the up-regulation of PI-3 kinase subunit p101 and p110γ phosphorylation *in vitro*^24^. Acute hyperglycemia in healthy obese women also induces an increase in the CD206 negative monocyte content and inflammatory markers such as CCL2 and TNF-α in subcutaneous abdominal adipose tissue^25^. Moreover, diabetic mice have elevated numbers of circulating monocytes and neutrophils owing to the hyperglycemia-induced proliferation and expansion of bone marrow myeloid progenitor cells^26^. However, the population size and function of hepatic monocytes/macrophages and neutrophils under hyperglycemic compared to euglycemic conditions are unknown. In this study, hepatic monocytes, but not macrophages, exhibited an increase in cell population size and in the expression of inflammatory cytokines in STZ-induced hyperglycemic mice (Figs 2 and 3). To exclude contamination by circulating monocytes from the peripheral blood in mice, we used a method based on *in situ* collagenase perfusion for the isolation of intact hepatic infiltrating monocytes and macrophages (Figs 2 and 3). Therefore, we investigated the cell-type specific gene expression in hepatic immune cells using flow cytometry-assisted cell sorting.

Tregs are essential for the regulation of excessive inflammatory responses in the body, but the population size and function of Tregs in hyperglycemia-induced inflammation is controversial. Some researchers have shown that the frequencies and functions of Tregs do not change or decrease in hyperglycemic NOD mice^27^,^28^, whereas STZ-induced hyperglycemia in mice leads to an increased Treg population from the thymus and enhanced generation of Tregs in the periphery^29^. In accordance with this previous report related to STZ-induced hyperglycemia, we showed that Tregs were significantly increased in the inflamed liver tissues and mesenteric lymph nodes from the diabetic mice. Moreover, in the current study, DEREG mice were prone to liver damage following STZ-induced hyperglycemia compared to the WT mice. This finding suggests that Tregs are critical for the control of hepatic inflammation in mice with STZ-induced hyperglycemia.

**Figure 6:**
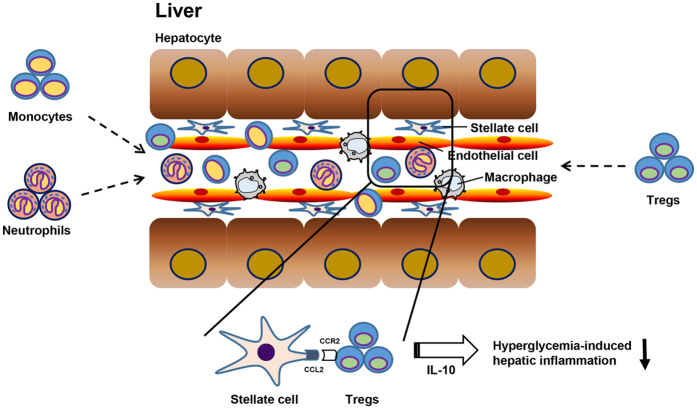
A summary figure depicting the infiltration of inflammatory cells and the protective role of Tregs in hyperglycemia-induced hepatic inflammation. The infiltration of monocytes and neutrophil facilitates inflammatory reactions in the liver in response to STZ-induced hyperglycemia, followed by the migration of CCR2^+^ Tregs into the inflamed liver, thereby improving hepatic inflammation.

Although CC chemokine receptors are essential for the trafficking and migration of Tregs into inflamed tissues or secondary lymphoid organs^30^,^31^,^32^,^33^, the hepatic migration of Tregs has not been elucidated in mice with STZ-induced hyperglycemia. In the previous study, CCR2^+^ Tregs were demonstrated to be important for the regulation of the inflammatory response in mice with collagen-induced arthritis, although CCR2 was essential for the recruitment of monocytes to sites of inflammation^34^. Additionally, CCR2 expression was required for the activation and migration of Tregs into draining lymph nodes in an islet allograft model^35^. CCR2-expressing Tregs also ameliorated pneumonitis and sialdenitis in MRL/lpr mice by accumulating in target organ^36^. Furthermore, we showed in the previous study that CCR2 was a critical factor for the migration of Tregs to HSCs in a T-cell mediated hepatitis mouse model^37^. In the current study, we revealed that CCR2 expression on Tregs was significantly increased in mice with STZ-induced hyperglycemia. HSCs but not hepatocytes or LSEC showed higher CCL2 expression (which is the ligand with the highest affinity for CCR2) in STZ-induced diabetic mice, suggesting the possible migration of CCR2^+^ Tregs into the hyperglycemia-induced inflamed liver (Fig. 6). Moreover, the treatment with STZ did not generate the induction of CCL2 expression of HSCs *in vitro* (Fig. 3e). This data suggests that CCL2 expression in HSCs might be regulated by inflamed environment in the liver by hyperglycemia rather than by STZ itself.

In this study, the migration of Tregs was also assessed using the *ex vivo* closed circulation technique explained in the Materials and Methods. This technique is quite simple but enables the analysis of the liver-specific migration of mouse cells in the circulating fluid through an infusion pump from the portal vein to the IVC. We expect that the closed circulation technique can be applied to research on hepatic sinusoidal endothelial shear stress by controlling the circulating flow velocity *ex vivo*.

In conclusion, our findings demonstrate that hepatic immune cells are activated in the livers of mice with STZ-induced hyperglycemia, thereby promoting liver inflammation. During the hyperglycemia-induced inflammatory process, Tregs are critical for immune homeostasis in the liver and CCR2 expression on Tregs is required for migration into the inflamed liver. Therefore, Treg-specific CCR2 agonism might be a therapeutic strategy for the treatment of liver damage in diabetes.

## Additional Information

**How to cite this article**: Lee, Y-S. *et al*. Hepatic immunophenotyping for streptozotocin-induced hyperglycemia in mice. *Sci. Rep*. **6**, 30656; doi: 10.1038/srep30656 (2016).

## Supplementary Material

Supplementary Information
