# Supplementary material for: Hepatic immunophenotyping for streptozotocin-induced hyperglycemia in mice
Source: Sci Rep. 2016 Jul 28;6:30656. doi: 10.1038/srep30656 (PMC4964583; doi:10.1038/srep30656)
Supplement: Supplementary Information [file srep30656-s1.pdf]

Hepatic immunophenotyping for streptozotocin-induced hyperglycemia in mice

Young-Sun Lee[1], Hyuk Soo Eun[2], So Yeon Kim[3], Jong-Min Jeong[3], Wonhyo Seo[3],

Jin-Seok Byun[4], Won-Il Jeong[5], Hyon-Seung Yi[2,5*]

[1]Department of Internal Medicine, Korea University College of Medicine, Seoul 136-705, Republic of Korea

[2]Department of Internal Medicine, Chungnam National University School of Medicine, Daejeon 305-764, Republic of Korea

[3]Laboratory of Liver Research, Biomedical Science and Engineering Interdisciplinary program, Korea Advanced Institute of Science and Technology, Daejeon 34141, Republic of Korea

[4]Department of Oral Medicine, School of Dentistry, Kyungpook National University, Daegu 41566, Republic of Korea

[5]Laboratory of Liver Research, Graduate School of Medical Science and Engineering, Korea Advanced Institute of Science and Technology, Daejeon 34141, Republic of Korea

[*]Corresponding author: Hyon-Seung Yi, M.D., Ph.D., Research center for endocrinology and metabolism, Chungnam National University Hospital, Munhwa-dong, Jung-gu, Daejeon 305-764, Korea Tel: 82-42-280-7140; Fax: 82-42-280-7995; Email: jmpbooks@cnuh.co.kr

Supplementary contents

1. Materials and methods
2. Supplementary figures
3. Supplementary table
4. References

Materials and methods

Liver TG assay

For measuring triglyceride, in liver, mice were sacrificed and their livers were removed and extracted. Livers were homogenized in 0.25% sucrose containing 1 mM EDTA. Lipids were extracted using chloroform/methanol (2:1), evaporated under dry nitrogen, and dissolved in 5% fatty acid-free BSA. Colorimetric triglyceride assays were carried out using the Sigma Diagnostics Cholesterol and Triglyceride Reagents (Sigma Chemical Co., St. Louis, MO, USA) [1].

Immunohistochemistry

Five-µm sections of paraffin embedded tissue blocks were prepared. Immunohistochemistry was performed with anti-F4/80 antibody (Cambridge, MA, USA). The antigen-antibody complexes were visualized with avidin-biotin peroxidase complex solution within an ABC kit (Vector Laboratories, Burlingame, CA) and DAB (Invitrogen, Eugene, OR, USA).

Staining

For hematoxylin and eosin staining in sorted monocytes and macrophages in the liver, the cells were attached to slide glasses by cytospin with 500 rpm for 5 minutes, fixed with 4% paraformaldehyde, permeabilized with 0.2% triton-X (Sigma-aldrich, St. Louis, MO, USA), then stained with hematoxylin and eosin as described previously [2].

Supplementary Figures and Supplementary Figure legends

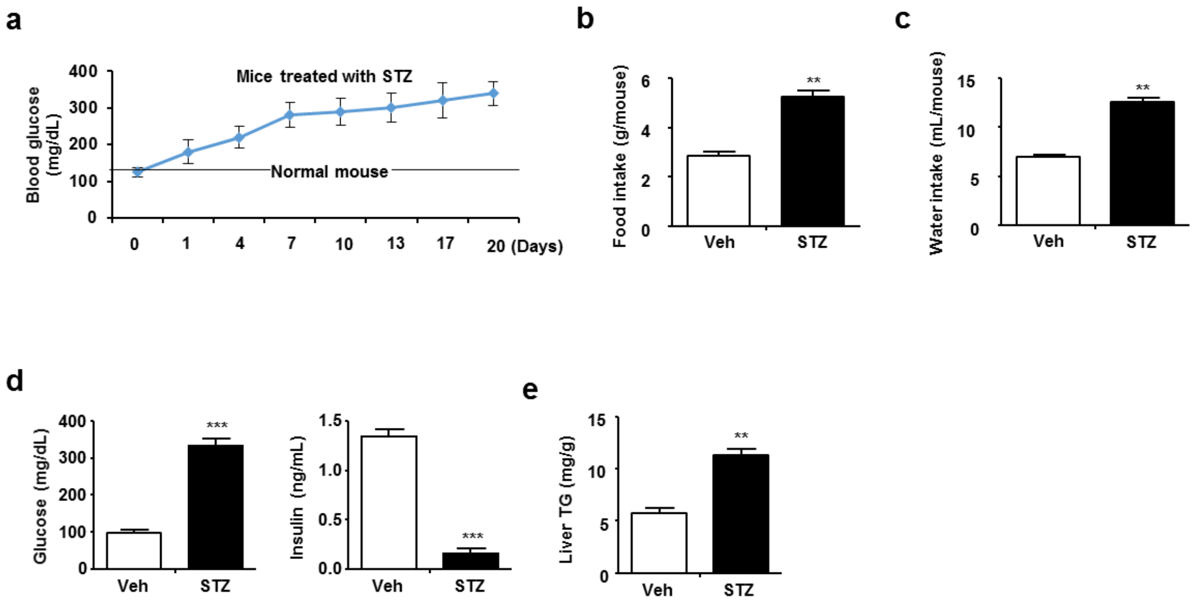

Supplementary Figure 1. Streptozotocin-induced hyperglycemia was generated in mice. (a) Glucose levels in STZ-induced hyperglycemic mice compared to normal mice. (b) Comparison of food intake between mice treated with the vehicle or STZ. (c) Comparison of water intake between mice treated with the vehicle or STZ. (d) Glucose and insulin levels in STZ-induced hyperglycemic mice. (e) Liver TG was measured in mice treated with the vehicle or STZ. All data are representative of 3 independent experiments (n = 6-8 per group). Data are expressed as the mean±SD. **P<0.01, ***P<0.001 compared with the corresponding controls.

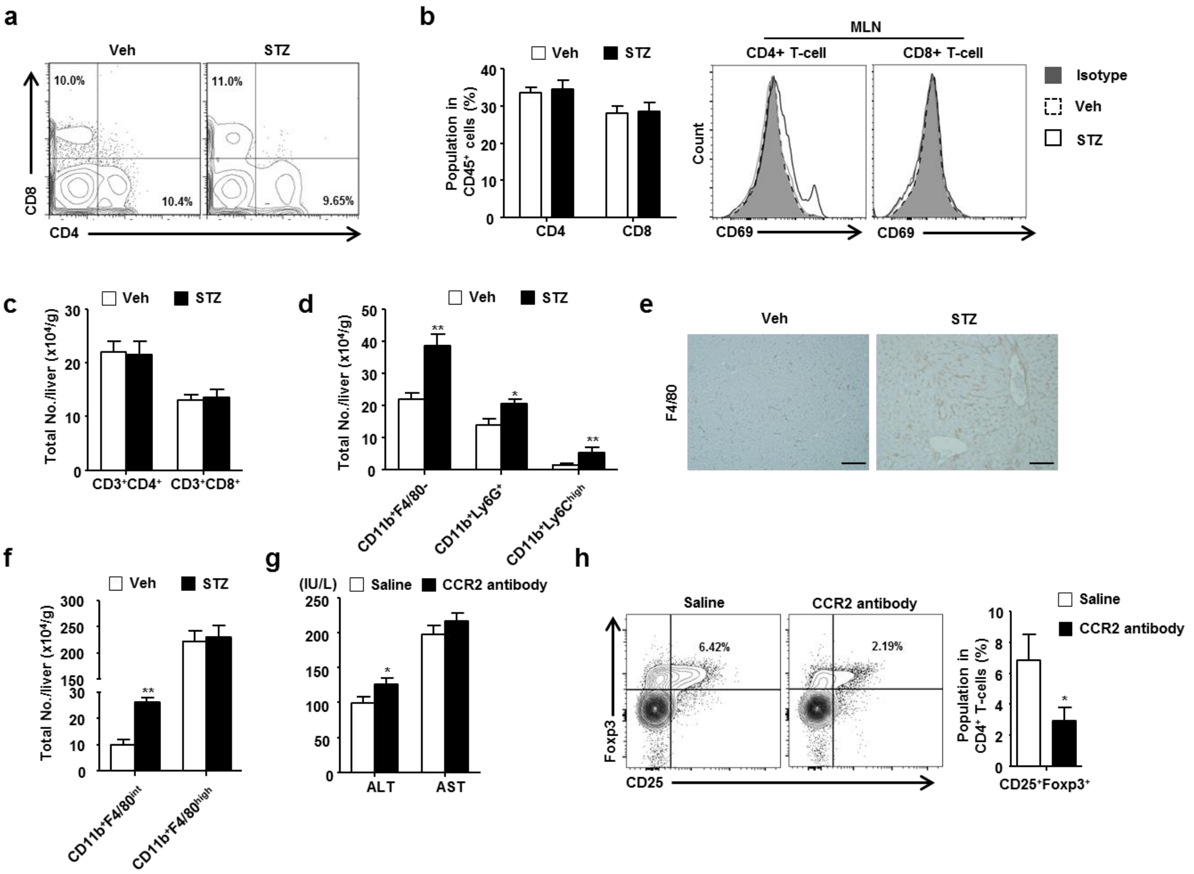

Supplementary Figure 2. Immuno-phenotype of mice with streptozotocin-induced hyperglycemia. (a) T cell population in mouse peripheral blood mononuclear cells. (b) T cell population in the livers of mice treated with the vehicle or STZ. (c,d) The absolute number of T cells, monocytes, and neutrophils in the liver from the mice treated with vehicle or STZ. (e) Liver sections were stained with the F4/80 antibody (original magnification, x100; scale bar = 200 μm). (f) The absolute number of hepatic monocytes and macrophages in the mice treated with vehicle or STZ. (g) Measurement of ALT and AST in the STZ-induced hyperglycemic mice treated with saline or CCR2 antibody. (h) The population of Treg in the liver from the STZ-induced hyperglycemic mice treated with saline or CCR2 antibody. All data are representative of 3 independent experiments (n = 6-8 per group). *P<0.05, **P<0.01 compared with the corresponding controls. Data are expressed as the mean±SD.

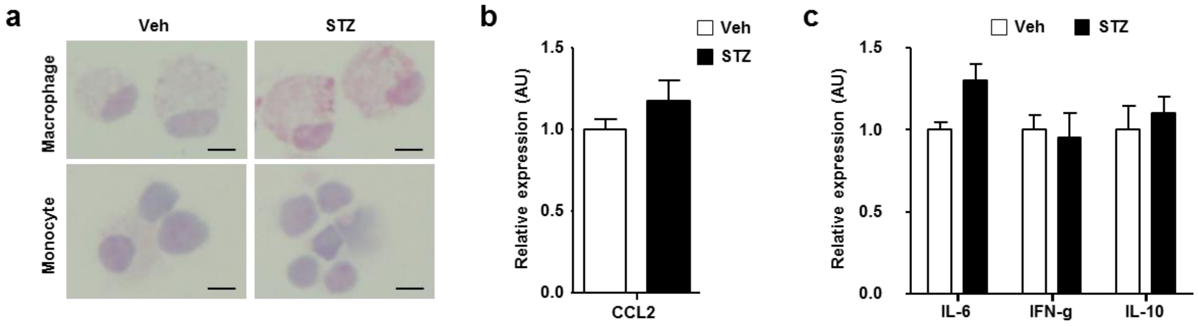

Supplementary Figure 3. Immune cells and liver sinusoidal endothelial cells of mice with streptozotocin-induced hyperglycemia. (a) Morphology of sorted hepatic monocytes and macrophages (original magnification, x200; scale bar = 10 μm). (b) CCL2 expression in the sorted hepatic sinusoidal endothelial cells from mice with vehicle or STZ. (c) Hepatic stellate cells were treated with STZ in vitro. All data are representative of 3 independent experiments (n = 6-8 per group). Data are expressed as the mean±SD.

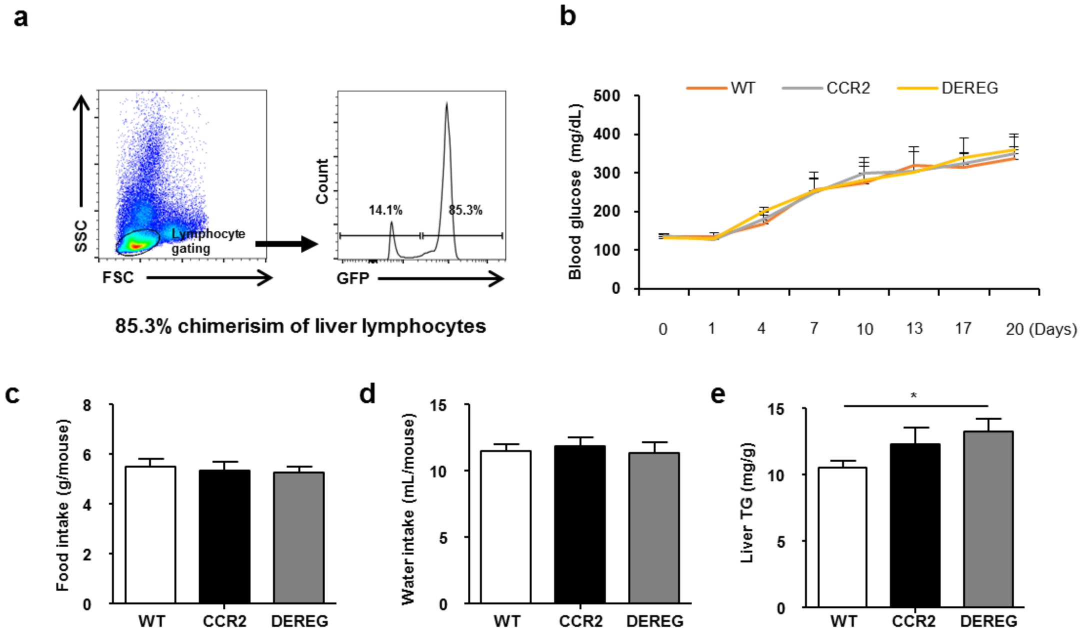

Supplementary Figure 4. Streptozotocin-induced hyperglycemia was generated in WT, CCR2 KO, and DEREG chimeric mice. (a) At week 8 after the transplantation of eGFP-producing WT bone marrow into WT mice, the chimerism of the hepatic immune cells was assessed by flow cytometry. (b) Glucose levels in WT, CCR2 KO, and DEREG chimeric mice with hyperglycemia. (c) Comparison of food intake between mice treated with the vehicle or STZ. (d) Comparison of water intake between mice treated with the vehicle or STZ. (e) Liver TG was measured in mice treated with the vehicle or STZ. All data are representative of 3 independent experiments (n = 6-8 per group). Data are expressed as the mean±SD. *P<0.05 compared with the corresponding controls.

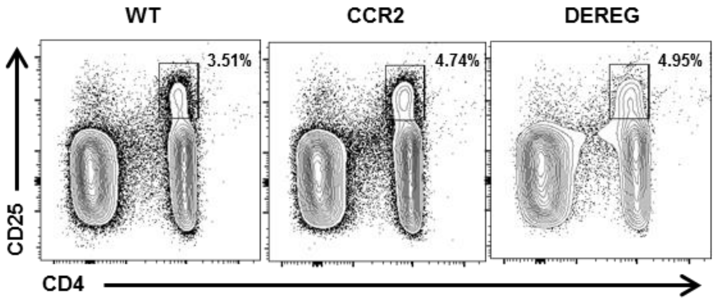

Supplementary Figure 5. Populations of hepatic CD4+CD25+ T cells in WT, CCR2 KO, and DEREG chimeric mice with hyperglycemia.

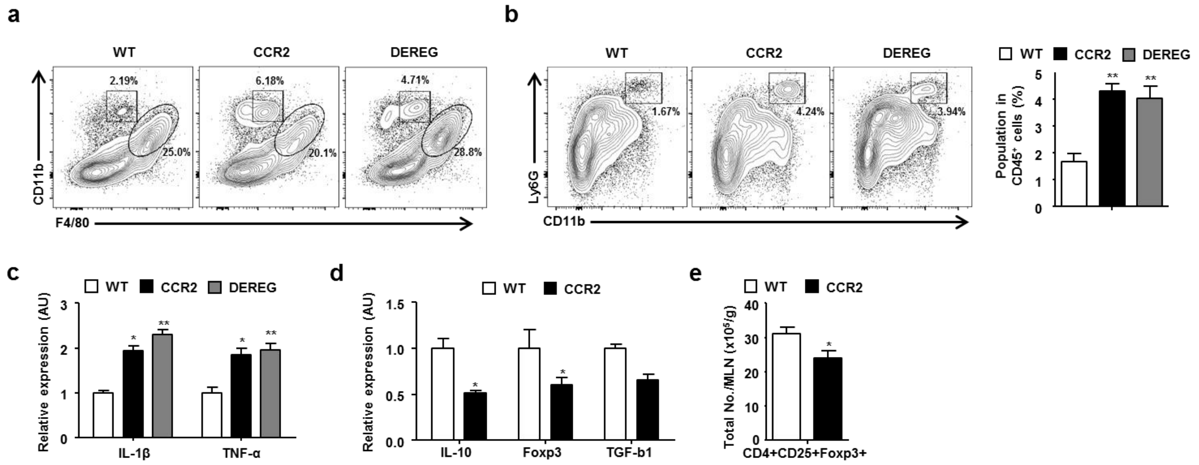

Supplementary Figure 6. Populations of hepatic immune cells in WT, CCR2 KO, and DEREG chimeric mice with hyperglycemia. (a) The populations of infiltrating monocytes and macrophages were analyzed using FACS. (b) The population of infiltrating neutrophils in the livers from the three groups of mice. (c) Infiltrating neutrophils in the liver were subjected to real-time PCR. (d) Tregs in the livers of WT and CCR2 chimeric mice were subjected to real-time PCR analysis. (e) The absolute number of Tregs in mesenteric lymph node from WT and CCR2 chimeric mice treated with STZ. All data are representative of 3 independent experiments (n = 6-8 per group). Data are expressed as the mean±SD. *P<0.05, **P<0.01 compared with the corresponding controls.

Supplementarly Table 1. Primers used in Real-time PCR (Mouse)

Genes	Forward (5'-3')	Reverse (5'-3')	PCR product (base pairs)
TNF-α	AAGCCTGTAGCCCACGTCGTA	AAGGTACAACCCATCGGCTGG	140
IL-1β	GCCCATCCTCTGTGACTCAT	AGGCCACAGGTATTTTGTCG	191
IFN-γ	AGACATCTCCTCCCATCAGCAG	TAGCCAAGACTGTGATTGCGG	182
IL-6	TCCATCCAGTTGCCTTCTTG	TTCCACGATTTCCCAGAGAAC	166
CCL2	TCAGCCAGATGCAGTTAACGC	TCTGGACCCATTCCTTCTTGG	184
CCR2	GGAGTGGGAAGAAGTATGT	TCAACCTTGGCAAGATAA	166
IL-10	GGTTGCCAAGCCTTATCGGA	ACCTGCTCCACTGCCTTGCT	196
Foxp3	CCCAGGAAAGACAGCAACCTTTT	GGCCTAAGGTCTTTCCATCC	219
TGF-β1	TTGCTTCAGCTCCACAGAGA	TTCTCACAACCAGGCCACTTG	88
β-actin	AGAGGGAAATCGTGCGTGAC	CAATAGTGATGACCTGGCCGT	148
GADPH	CGTGCCGCCTGGAGAAACC	TGGAAGAGTGGGAGTTGCTGTTG	144

References

[1] You M, Matsumoto M, Pacold CM, Cho WK, Crabb DW. The role of AMP-activated protein kinase in the action of ethanol in the liver. Gastroenterology 2004;127:1798-1808.

[2] Suh YG, Kim JK, Byun JS, Yi HS, Lee YS, Eun HS, et al. CD11b(+) Gr1(+) bone marrow cells ameliorate liver fibrosis by producing interleukin-10 in mice. Hepatology 2012;56:1902-1912.
